# Effect of bioactive glass paste on efficacy and post-operative sensitivity associated with at-home bleaching using 20% carbamide peroxide: a randomized controlled clinical trial

**DOI:** 10.1186/s40001-022-00826-5

**Published:** 2022-10-04

**Authors:** Yazan Bizreh, Hussam Milly

**Affiliations:** Department of Restorative Dentistry, Faculty of Dental Medicine, Damascus University, Mazah, Damascus, Syria

**Keywords:** Bleaching, Bioactive glass 45S5, Sensitivity, Colour, Carbamide peroxide

## Abstract

**Background:**

The aim of this study was to evaluate the effect of bioactive glass (BAG) 45S5 paste on colour change and tooth sensitivity (TS) when used in combination with 20% carbamide peroxide (CP) during at-home vital tooth bleaching.

**Methods:**

Twenty-four patients were selected and assigned into two experimental groups (*n* = 12) in a double-blind study design. Each patient received 20% CP followed by the application of either BAG paste or non-active placebo paste. The shade evaluation was performed using a digital spectrophotometer based on the CIE L*a*b* colour space system at different time points and the overall colour changes ΔE were calculated. TS was evaluated using visual analogue scale (VAS). The values of ΔE and TS were statistically analysed using paired *t*-test. The level of statistical significance was established at *p* = 0.05.

**Results:**

The overall colour changes (ΔE) between baseline and each time point showed no significant differences between BAG and placebo groups (*p* > 0.05). The use of BAG paste significantly decreased TS reported by the participants.

**Conclusions:**

The association of BAG paste with at-home bleaching treatment presents a promising method as it decreased TS and did not deteriorate bleaching efficacy.

*Trial registration* This study was approved and registered in the Australian New Zealand Clinical Trials Registry (ANZCTR) under Registration number: ACTRN12621001334897.

## Background

Teeth discoloration is one of the most common aesthetic concerns, and several efforts have been made to develop conservative treatments to deal with this aesthetic problem [1, 2]. Teeth bleaching presents a safe approach to manage teeth discoloration with minimal destructive procedures in comparison with alternative techniques such as porcelain crowns and veneers [3, 4]. Various protocols for vital teeth bleaching were introduced including at-home bleaching which utilizes different concentrations of carbamide peroxide (CP). In this technique, the bleaching gel is applied by patients themselves using a custom tray for a period depending on CP concentration [2, 5, 6]. Undesirable side effects, however, were reported when CP was used in teeth bleaching such as gingival irritation, morphological/chemical alterations in tooth structures, deteriorative effects on restorative materials and teeth sensitivity (TS) [7–9].

Teeth sensitivity is a common side effect that initiates immediately after bleaching commencement and many factors contribute in the aetiology and progression of TS [3]. The patients suffer from abrupt, spontaneous, intense pain in single or multiple teeth and might be forced to stop bleaching in some cases when TS becomes severely discomfort [3, 10, 11]. This side effect has been documented frequently in the literature and the incidence ratio varies between 37 and 90%, according to previous investigations [8, 12, 13].

Bioactive glass 45S5 (BAG), invented in 1969 by professor Larry Hench [14, 15], has been employed in various applications in dentistry due to its bioactive properties and its capability to bond with hard and soft body tissues [16, 17].

It consists of 45% SiO_2_, 24.5% Na_2_O, 24.5% CaO, and 6% P_2_O_5_ [18]. BAG particles release calcium, phosphate, and sodium ions in the oral environment when immersed in aqueous solutions such as saliva [19, 20]. This bioactivity rises the pH, forms a hydroxycarbonate apatite (HCA) layer and enhances enamel remineralization [20, 21]. It has been stated that BAG with ultra-fine particles (< 20 μm) can significantly reduce TS by occluding the openings of dentinal tubules with HCA depositions and therefore, it was introduced as an active component in some commercial sensitive relief pastes [22].

Since there is no previous controlled clinical trial reported in the literature evaluating the potential role of BAG in reducing TS reported with vital at-home teeth bleaching, the aim of this double-blind randomized, controlled clinical trial was to investigate the effect of BAG based pastes on colour change and TS associated with at-home vital teeth bleaching using 20% CP. The null hypotheses investigated were that BAG paste had no effect on TS associated with the use 20% CP bleaching gel and that BAG paste would not affect bleaching efficacy.

## Materials and methods

### Ethical approval and protocol registration

This double-blind randomized, parallel group controlled clinical trial was approved and registered in the Australian New Zealand Clinical Trials Registry (ANZCTR) under Registration number: ACTRN12621001334897. This study follows the Consolidated Standards of Reporting Trials (CONSORT) statement [23].

### Sample size and recruitment

The sample size was calculated using GPower 3.1 software. Effect size (d) for colour change ΔE was considered for the calculation of the sample size. Based on a previous study the effect size (d) for colour change was 1.98 [24]. Using *t*-Student test for two independent samples with a statistical power of 95% and a significance level 5%, It was necessary to enrol 16 patients (eight participants per group) in this superiority trial. Twelve participants per group were recruited, taking into consideration potential loss for follow-up with an overall sample size *n* = 24. All volunteers participated in this study signed informed consent after a thorough explanation of this investigation.

### Eligibility criteria, randomization and blinding

The participants were required to have at least six maxillary anterior teeth free of restorations or carious lesions on their buccal surfaces with colour shade A2 or darker on the shade guide. Participants included in this study should have good general and oral health.

The exclusion criteria included participants with a history of tooth sensitivity, using desensitizing agent/paste in the past 3 months, pregnant/lactating women, patients with chronic therapeutic drug history and smokers.

Patients who were using orthodontic appliances, had bleached their teeth previously, had severe tooth discoloration or allergies to the materials used in the study were excluded as well.

In this double-blind clinical trial, both the participants and the examiner were masked to the group assignment. Participants were randomly assigned into two experimental groups (*n* = 12) by dragging a paper that showed the code of the paste used after bleaching procedures either BAG (A) or Placebo (B) pastes. Both pastes were delivered in similar syringes coded as “A” and “B”, with the same texture and colour. A staff member, who was not involved in the evaluation process, was responsible for randomization, allocation and paste syringes coding.

### Intervention

Two custom trays were prepared for each participant using soft vinyl sheets, 0.8 mm (Sof-Tray Classic, Ultradent, South Jordan, UT, USA) and trimmed 1 mm beyond the gingival margins. The first custom tray was made for at-home bleaching procedure, whilst the second one was provided with holes in the middle of the buccal surface of the anterior teeth for spectrophotometer measurements in subsequent follow-up sessions [6, 25–28]. Each patient was provided with a kit containing: (1) bleaching tray, (2) the bleaching gel syringe (Opalescence^®^ PF™, Ultradent Products Inc. USA—20% carbamide peroxide), (3) coded container containing either bioactive glass paste (Sensodyne Repair & Protect contains BAG 45S5: 5.0% w/w active ingredient) or non-active placebo paste and (4) oral hygiene kit including toothbrush and non-whitening paste in order to standardize daily oral hygiene protocol for all subjects.

All participants received a practical demonstration and a leaflet with instructions regarding the application of the bleaching gel and the experimental paste. They were asked to apply the bleaching gel for 4 h using the bleaching tray followed by rinsing the teeth and the tray to end with the application of the experimental paste in the same bleaching tray for 30 min daily for 7 days [24].

### Outcomes

#### Colour change evaluation

The experimental unit consisted of 48 teeth, and the shade evaluation was performed for both upper left central incisor and upper right canine using Easy Shade Advance 4.0 spectrophotometer (VITA Zahnfabrik, Bad Säckingen, Germany) at seven different time points: T_0_ before treatment (baseline), T_1_ after 3 days, T_2_ after one week, T_3_ after two weeks, T_4_ after one month, T_5_ after 3 months, T_6_ after 6 months. After calibration of the spectrophotometer, its tip was inserted in the holes of the positioning guide tray to obtain the shade based on the CIE L*a*b* colour space system, measurements was repeated three times. The colour difference (ΔE) between baseline and each time point was calculated using the following formula [29]:

$$ \Delta {\text{E}}^{*} = \left\{ {\left( {\Delta {\text{L}}^{*} } \right)^{2} + \left( {\Delta {\text{a}}^{*} } \right)^{2} + \left( {\Delta {\text{b}}^{*} } \right)^{2} } \right\}^{{{1 \mathord{\left/ {\vphantom {1 2}} \right. \kern-\nulldelimiterspace} 2}}} . $$

#### Tooth sensitivity evaluation

Patients were instructed to assess TS daily for 7 days using visual analogue scale (VAS) immediately after waking up to standardize the assessment time. Visual analogue scale is a numeric scale graded from 0 which means no TS to 10 which means severe TS. In addition, patients were asked if they had experienced any TS incidence during the 6-month period following the bleaching procedure [30].

### Statistical analysis

Kolmogorov- distributed test was conducted to evalute normal distribution using SPSS^®^ statistical software V22 (spss inc. Chicago il USA). Colour change ΔE and TS values were analysed using paired t-test. The level of significance for all tests was 5%.

## Results

**Fig. 1:**
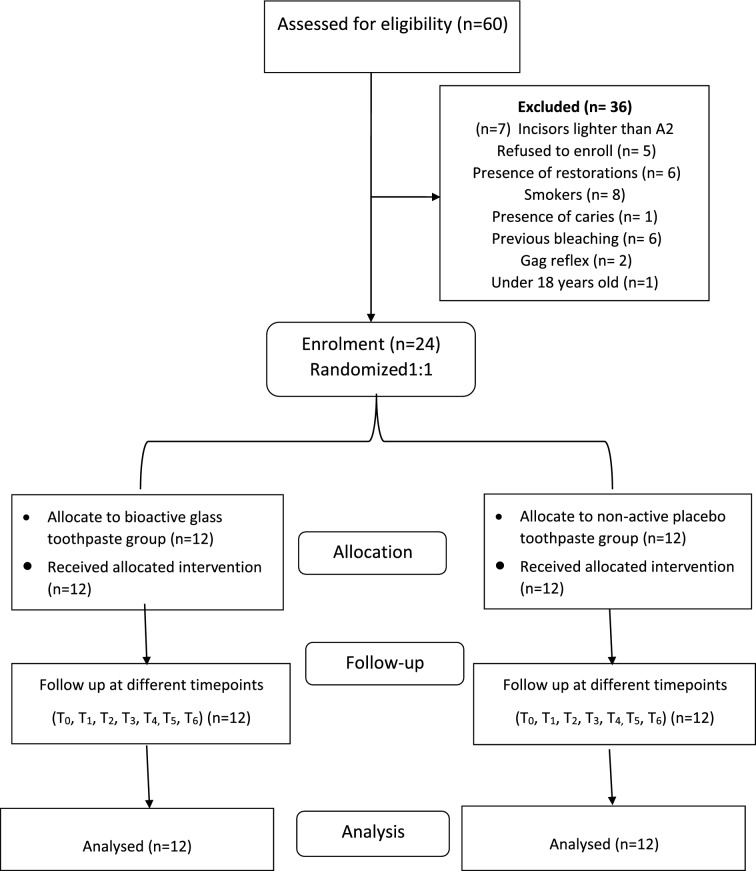
CONSORT flow diagram of the study

A total of 60 participants were examined and 24 participants were enrolled in this trial (Fig. 1). Fourteen participants were female (58.3%) and 12 were male (41.7%), having an age range between 18 and 28 years (23.1 ± 2.6, mean ± SD).

**Fig. 2:**
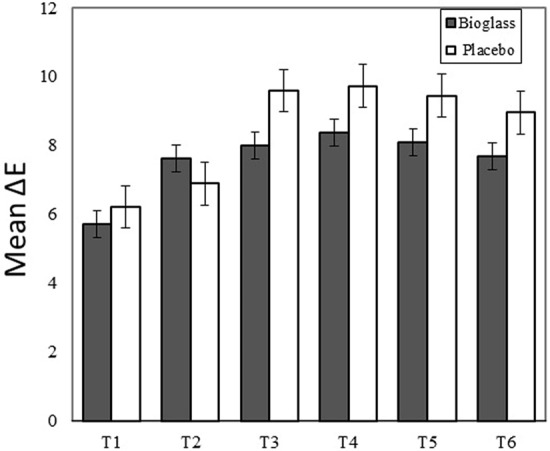
ΔE (means ± SE) in the BAG and placebo groups

Figure 2 and Table 1 present mean ± SE of the ΔE values in the BAG and placebo groups. There were no significant differences between BAG and placebo groups at any measurement point (*p* > 0.05). The ΔE values exhibited statistical differences within BAG groups between T1 time point vs each other time point as follows: T2: *p* = 0.006, T3: *p* = 0.008, T4: *p* = 0.001, T5: *p* = 0.002, T6: *p* = 0.008. In the placebo groups, the ΔE values between T1 vs time points (T3, T4, T5, T6) and between T2 vs (T3, T4, T5, T6) time points, showed significant differences *p* < 0.001. No significant difference was record between T1 and T2 in placebo group (*p* > 0.05).

**Table 1:** ΔE values at different time points within each group

Time	Group	Mean ΔE	SE	*P*
T1	BAG	5.72	0.54	0.530
	PLA	6.22	0.56	
T2	BAG	7.63	0.73	0.437
	PLA	6.90	0.57	
T3	BAG	8.00	0.69	0.095
	PLA	9.60	0.63	
T4	BAG	8.38	0.62	0.120
	PLA	9.74	0.59	
T5	BAG	8.10	0.74	0.169
	PLA	9.45	0.63	
T6	BAG	7.70	0.77	0.246
	PLA	8.97	0.75	

Differences between the groups were evaluated using *T*-test and considered as statistically significant at *p* < 0.05

Overall, the use of BAG decreased TS when compared with the placebo group (*p* = 0.013) (Table 2). The BAG use during at-home bleaching reduced significantly TS reported by the patients at the first day when compared with the placebo group. The TS was less in the BAG group after 3 days than those reported by the participants in the placebo group. Likewise, TS described by patients in the BAG group was less than the placebo group after 6 days but without any statistical difference. In the 6-month follow-up period, only three participants in the placebo group still reported TS incidence.

**Table 2:** TS reported by patients using VAS

Time	Group	TS Mean	SE	*P*
Day-1	BAG	0.33	1.88	0.039
	PLA	1.08	0.609	
Day-3	BAG	0.75	0.329	0.109
	PLA	1.67	0.667	
Day-6	BAG	0.67	0.310	0.096
	PLA	1.67	0.620	
Overall	BAG	0.79	0.131	0.013
	PLA	1.42	0.214	

Differences between the groups were evaluated using *T*-test and considered as statistically significant at *p* < 0.05

## Discussion

At-home or dentist-supervised night-guard bleaching has been considered the gold standard technique for vital teeth bleaching since it needs less chair time and has minimal side effects compared to the other techniques [31, 32]. Nevertheless, several studies have reported TS incidence with at-home bleaching which varies according to different factors such as the concentration of CP and the exposure time [8, 12, 33, 34]. The use of CP leads to reduced enamel microhardness and increased surface roughness [21, 35–38]. The peroxide penetration into the pulp in conjunction with the increasing of enamel/dentine permeability initiates TS, reported during teeth bleaching [38].

Different materials and pastes have been introduced to reduce TS during teeth bleaching, [7, 21, 24, 38–43]. These agents may reduce sensory nerve activity such as potassium salts or block dentine tubules as fluoride, arginine, CPP-ACP and BAG [39, 44]. The results of the present study demonstrated that the use of BAG paste significantly reduced TS and therefore, the first null hypothesis was rejected.

The BAG mechanism in decreasing TS might be explained due to its remineralization effect and the deposition of the hydroxycarbonate apatite (HCA) layer which has a role in occluding dentinal tubules [45].This is in agreement with a previous clinical study where the application of BAG decreased significantly the TS associated with in-office bleaching using 35% hydrogen peroxide (HP) gel [46]. A beneficial effect for BAG pastes (2.5–7.5%) in reducing TS was documented in a previous systematic review [44]. The evaluation of the possible impact of BAG pastes on TS related to at-home tooth bleaching with 20% CP has not been reported in the literature.

The second null hypothesis investigated in this trial was accepted as the statistical analysis revealed that the application of bioactive glass during the bleaching procedure has no effect on bleaching efficacy. A previous clinical trial showed that the utilizing of either CPP-ACPF or BAG did not compromise tooth bleaching with 35% hydrogen peroxide [41]. Several in vitro studies demonstrated that the application of BAG either before, after or during teeth bleaching did not negatively affect the bleaching efficacy. Moreover, this association exhibited many benefits including, reduced mineral degradation [47, 48], reduced losing of enamel microhardness [49, 50], conserved enamel surface integrity [50, 51] and minimized the infiltration of peroxides into pulp chambers [52].

In the present study, the experimental pastes were applied using the custom transparent bleaching tray daily following bleaching procedure to guarantee sufficient and standardized exposure time [24, 53]. Previous in vitro study demonstrated that BAG paste led to a better formation of the HCA layer and more efficient dentinal tubules occlusion when dispensed with transparent tray as this technique prevents dilution of BAG paste by oral fluids [52]. Further investigations are still required to ascertain the beneficial effect of BAG on reducing TS when various concentrations of CP and HP are used for both at-home and in-office bleaching procedures.

## Conclusion

The association of BAG with at-home bleaching presents a promising method to reduce TS reported by the patients without any deteriorating effect on bleaching efficiency.

## Data Availability

The datasets used and/or analysed during the current study are available from the corresponding author on reasonable request.

## Abbreviations

BAG: Bioactive glass
TS: Tooth sensitivity
CP: Carbamide peroxide
HCA: Hydroxycarbonate apatite
VAS: Visual analogue scale
HP: Hydrogen peroxide
ANZCTR: Australian New Zealand Clinical Trials Registry
CONSORT: Consolidated Standards of Reporting Trials
